# Correction: Zero valent iron/poly(amidoxime) adsorbent for the separation and reduction of U(vi)

**DOI:** 10.1039/d6ra90031c

**Published:** 2026-03-26

**Authors:** Dadong Shao, Xiangxue Wang, Xiaolin Wang, Sheng Hu, Tasawar Hayat, Ahmed Alsaedi, Jiaxing Li, Suhua Wang, Jun Hu, Xiangke Wang

**Affiliations:** a Institute of Nuclear Physics and Chemistry, China Academy of Engineering Physics Mianyang 621900 China xlwang@caep.ac.cn; b Collaborative Innovation Center of Radiation Medicine of Jiangsu Higher Education Institutions, School for Radiological and Interdisciplinary Sciences, Soochow University Suzhou 215123 P.R. China xkwang@ipp.ac.cn; c NAAM Research Group, Faculty of Science, King Abdulaziz University Jeddah 21589 Saudi Arabia; d Department of Mathematics, Quaid-I-Azam University Islamabad 44000 Pakistan; e School of Environment and Chemical Engineering, North China Electric Power University Beijing 102206 China

## Abstract

Correction for ‘Zero valent iron/poly(amidoxime) adsorbent for the separation and reduction of U(vi)’ by Dadong Shao *et al.*, *RSC Adv.*, 2016, **6**, 52076–52081, https://doi.org/10.1039/c6ra10817b.

The authors regret that the SEM images of ZVI/PAO in Fig. 1 were incorrect. The experiment was repeated and the corrected Fig. 1C and D are shown herein.

**Fig. 1:**
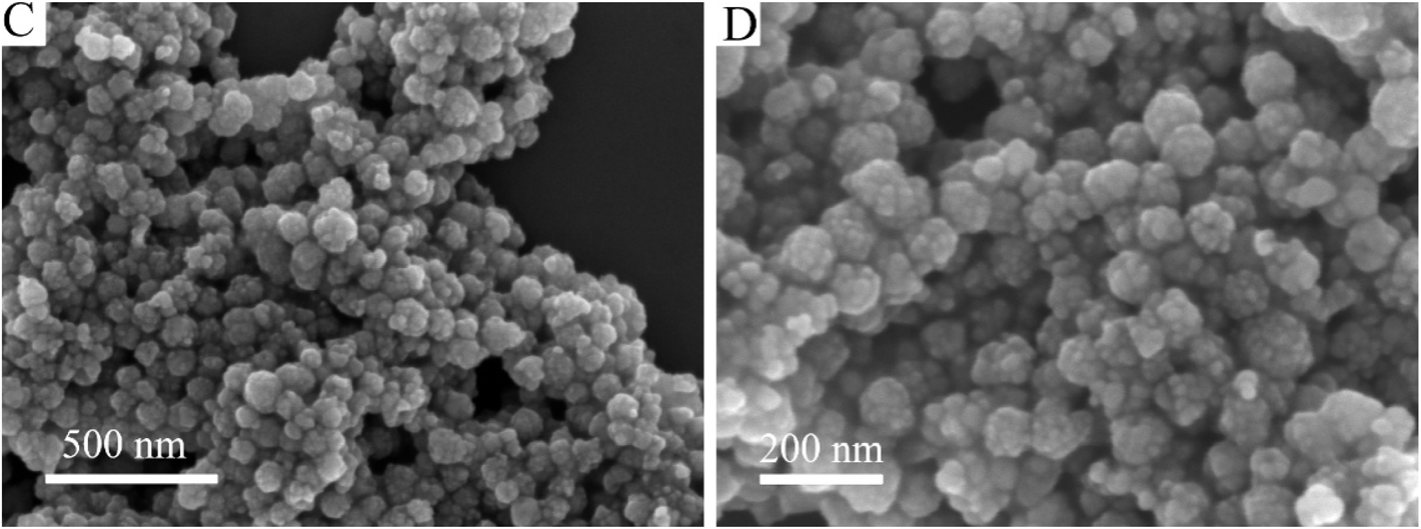
SEM images of ZVI/PAO (C and D).

An independent expert has viewed the corrected Fig. 1 and confirmed that it is consistent with the discussions and conclusions presented.

The authors sincerely apologise and acknowledge the error, and thank the reader for their important PubPeer comment (https://pubpeer.com/publications/069B0B5D7CF8C7472635ACED1EE700).

The Royal Society of Chemistry apologises for these errors and any consequent inconvenience to authors and readers.

